# “I’ll leave that to the case managers.” Healthcare Service Providers‘ Perceptions of Organizational Readiness for Change in a Randomized Controlled Trial—A Qualitative Analysis Exploring Implementation Success

**DOI:** 10.3390/ijerph19095782

**Published:** 2022-05-09

**Authors:** Kyung-Eun (Anna) Choi, Lara Lindert, Lara Schlomann, Holger Pfaff

**Affiliations:** 1 Center for Health Services Research, Brandenburg Medical School Theodor Fontane, Fehrbelliner Str. 38, 16816 Neuruppin, Germany; lara.lindert@mhb-fontane.de; 2 Institute of Medical Sociology, Health Services Research and Rehabilitation Science, Faculty of Medicine, University Hospital Cologne, Faculty of Human Sciences, University of Cologne, Eupener Str. 129, 50933 Cologne, Germany; lara.schlomann@uk-koeln.de (L.S.); holger.pfaff@uk-koeln.de (H.P.); 3 Health Services Research, MIAAI group, Faculty of Medicine/Dentistry, Danube Private University, Steiner Landstr. 124, 3500 Krems an der Donau, Austria

**Keywords:** organizational change, readiness for change, employee health, sick leave, return to work, prevention, rehabilitation, case management, implementation, musculoskeletal disorders

## Abstract

Up to 50% of unsuccessful implementations of organizational change are due to a lack of organizational readiness for change (ORC). This qualitative study aims to investigate the experiences of occupational physicians (OPs) and staff of test and training centers (ETTCs) with team effectiveness in the context of ORC. The change setting is the implementation of a new occupational health program in a multicentric randomized controlled trial for musculoskeletal disorders (MSD) in Germany. Two rounds of expert interviews with OPs (1st round: *n* = 10, 2nd round: *n* = 13) and one round of expert interviews with ETTCs (*n* = 9) were conducted and analyzed with a deductive–inductive procedure. The focus of the analysis was the assessment of change commitment and change efficacy, as well as their influence on general ORC on a collective level according to Weiner’s model (2009). Differential critical assessment of change by the care providers led to a missing collective change commitment and consequently to a missing organizational change commitment. Main inhibiting factors include lacking feedback about (e.g., recruitment) success, limited time resources of and narrow communication between responsible study staff, along with a low rate of utilization and limited adherence of the study population. Main facilitators include standardized procedures and documentation along with easy-access digital tools. Researchers may use the findings to improve the development of new intervention studies, especially in a randomized setting.

## 1. Introduction

Healthcare systems are exposed to constant change, at some points in disruptive waves due to recent public health policy, altered demands of the market, and technological development as seen in digitalization [1]. Successful implementations of new health-promoting programs are complex procedures and demand the coordinated cooperation of multiple partners [2]. This concerns not only the introduction of new evidence and guidelines into routine daily practice, but especially the scientific evaluation of new care models in real-world settings with randomized controlled trials (RCTs). Typically, these trials compare the effects of an assumed superior treatment to a control intervention. In unblinded settings, the implementation process comprises not only the introduction, the adoption, and the maintenance of the relevant new content, but also a critical handling of the alleged inferior control intervention.

Implementation processes are per se demanding: the reorganization of routines and the maintenance of an efficient communication flow demand additional time and cognitive resources of all parties involved. Up to 50% of unsuccessful implementations of organizational change are due to a lack of organizational readiness for change (ORC) [3].

### 1.1. Organizational Readiness for Change

Previous definitions of ORC describe it as “a comprehensive attitude simultaneously by the nature of the change, the change process, the organization’s context, and the attributes of individuals” [1,4]. Change management research emphasizes the pivotal role of organizational readiness as a prerequisite for successful change implementation [3], especially in highly complex systems such as healthcare. Over the past 20 years, the concept of ORC has received increasing attention in healthcare management [1], with a growing amount of empirical research on ORC [5,6]. However, there is not a consensus on the core theoretical components of ORC in general or of ORC for healthcare systems in particular, yet.

According to Weiner, ORC is an interplay between the organizational members’ determination to change (change commitment) and “their shared belief in their collective capability to do so (change efficacy)” [3]. The more that organizational members believe that the envisioned change is valuable, the more they will engage in the change implementation process [3]. To achieve this commitment, a prerequisite is to fully understand the extent of the targeted aims. Only then, it is possible for all members to judge if all necessary information and resources are available. A positive evaluation on these questions will strengthen the common belief in their collective capability and will lead to a high change efficacy [3]. Broader context factors may also influence organizational readiness for change. These may include a general organizational culture that is open to taking risks and embracing innovations or a shared narrative of positive past changes that led to organizational success [3]. ORC might support the closure of the evidence–practice gap in knowledge translation efforts, when scientific evidence is transferred to everyday healthcare [7].

### 1.2. Creating New Networks

Multicenter trials investigating new healthcare programs underlie dynamic, complex forces comprising knowledge creation and application to foster the network. Individual strategies are often condemned to fail; moreover, a joint action for implementation is needed considering contextual or organizational barriers and using all available facilitators [8]. Especially in new study settings, when the different parties do not share a common work history yet, it is very difficult to implement new pathways and foster teamwork and shared actions. Guidelines of organizational and human resources development recommend consistent leadership actions/messages and the extensive sharing of relevant information and common experiences, including experience with past efforts with other changes [6,9]. An analysis of the main facilitators and barriers of implementation supports the critical assessment of its potential sustainability and appraises at least task demands, resource availability, and situational factors. According to Weiner [3], the concept of ORC can be used to assess the organizational capacity to engage in substantial changes.

In summary, ORC can serve as a key overarching theoretical concept to evaluate organizational members’ collective motivation, willingness, and capability to implement change. In this qualitative study, we therefore use ORC as a concept to map the experiences of occupational physicians (OPs) and staff of test and/or training centers (ETTCs) as key network partners and service providers within a newly created network to implement an innovative healthcare program for musculoskeletal disorders [10]. The overarching aim of the formative evaluation within the trial was to identify specific barriers and facilitators of implementation processes.

Our main research question (RQ) for the following analysis is:

RQ: How do they perceive the implementation process and how do their perceptions relate to the network’s organizational readiness for change?

We hypothesized that an organization with high organizational readiness for change as a prerequisite would perceive less barriers and more facilitators in the implementation of the new program.

## 2. Materials and Methods

The current study is part of the formative evaluation of a new modular, workplace-related, occupational health program in the setting of a multicentric randomized controlled trial (RCT) for musculoskeletal disorders (MSD) in Germany [10]. The four-year trial investigated the effects of a coordinating case management against a supported self-management concept, which was based on German standard care for MSD. The study network included 22 German companies (mainly steel and metal manufacturing, automotive industry, trade, and service), 12 pension funds, 15 company health insurance funds with more than 44,000 insured employees, OPs, ETTCs, and rehabilitation facilities. Former analyses by our group identified study challenges from case managers’ perspectives [11], as well as the utilization behavior characteristics of study participants [12]. Besides the summative evaluation of the predefined primary outcomes of sick leave days, workability, perceived pain, and self-efficacy [10], the main trial also used a set of qualitative methods in different formats (focus groups, telephone interviews) to investigate the implementation process in a formative evaluation in a more open-investigative manner. A predefined set of barriers and facilitators would have restricted us too much, since there are too many competing valuable concepts and measures in assessing too many different facets, such as collaboration, team effectiveness, trust, and support, to name only a few. So we decided to follow an open, explorative approach.

The current analysis is part of its formative/process evaluation and adheres to the consolidated criteria for reporting qualitative studies (COREQ) guidelines [13].

### 2.1. Accessing the Sample

To evaluate the implementation process and to identify facilitators and barriers from different perspectives, we interviewed Ops in 2018 and 2019, as well as ETTCs in 2020. All Ops and ETTCs with duties in the RCT were invited to share their experiences and views on the implementation via telephone expert interviews. To take part in the overarching trial, all registered company insurance funds were invited with their cooperating partners. The resulting network included small- to large-sized companies and insurance funds with diverse structures concerning employees and insured. The initial invitations were sent via email. Subsequently, potential study participants were contacted by phone. Interested OPs and ETTCs received a standardized short questionnaire on sociodemographic details. All study participants gave their written informed consent.

### 2.2. Setting Procedure and Data Collection

All topic lists aimed at discovering the barriers and facilitators of program implementation. Due to the specific tasks and duties for OPs and ETTCs, respectively, the interview guides were adapted to their responsibilities. The semi-structured interview guides were designed by the research team by considering major aspects of the Consolidated Framework for Implementation Research (CFIR) [14] as well as current project developments, assessed via feedback in project meetings of the wider network. Potential interviewees were approached by members of the study team. In each interview, additional field notes were made by the interviewer. The telephone interviews were collected by three independent interviewers (L.L., L.N. and L.S.) during the working hours of the interview partners. Employers were informed about the procedure and gave consent for the data collection. Interviewers had interdisciplinary backgrounds (health economics, sociology, and rehabilitation sciences), prior experience with qualitative research, and were all female researchers. Study participants were familiar with the interviewers from previous telephone/mail contact. During the data collection, no one else was present.

### 2.3. Data Analysis

All telephone interviews were audio-recorded, transcribed verbatim, and pseudonymized. Data analysis followed a deductive–inductive approach. We mapped the study network as an organization. According to Frese and colleagues, organizations are characterized by (a) the involvement of several involved actors and their (single as well as collective) actions, and (b) the joint orientation towards a shared aim [15]. In their model, the single actions of one individual can influence the actions of others.

**Figure 1:**
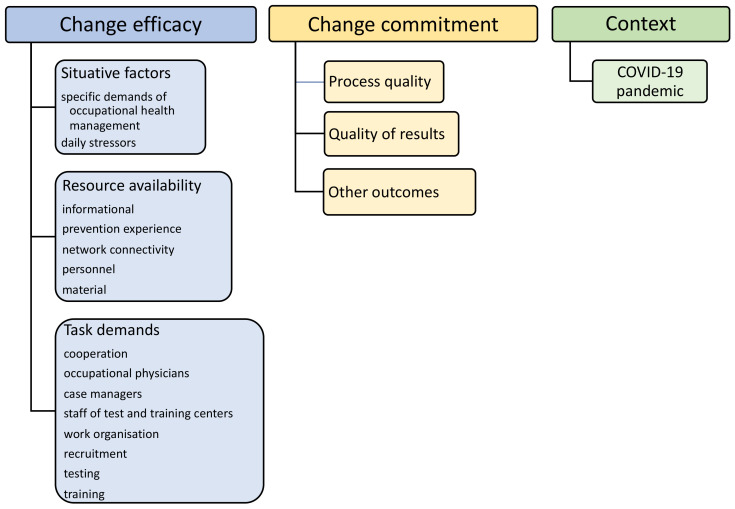
Coding scheme.

Category schemes were theoretically driven and encompassed three main categories as key components of Weiner’s [3] readiness for change model: (1) change efficacy (with subcategories/indicators: (a) resource availability, (b) situative factors, and (c) task demands); (2) change commitment (with subcategories/indicators: (a) process quality, and (b) quality of results), as well as (3) context factors (see Figure 1). We used definitions of the model’s components to map and frame discovered themes in the analysis. Herein, we followed a qualitative content analysis scheme according to Gläser and Laudel [16]. Additional subcategories were added using the material (see Figure 1 for the final coding scheme). Inductively generated categories were reviewed within the group and added to the coding scheme in an iterative procedure. We repeatedly discussed and reviewed any modification to the coding scheme within the research team.

Data were coded using MAXQDA 2020. Initially, one interview was coded by two researchers together to check for mutual understanding of the overall coding scheme and overlapping of codes. Repeated discussions and reflections guided us to solve any uncertainties. Finally, one researcher repeated the coding process for all transcripts with the finalized coding scheme under supervision of a second senior researcher. The use of field notes and several rounds of peer review ensured the intersubjectivity of the results and minimized possible bias in data analysis. Quotes representative of the results were pre-selected by one coder each and then agreed on in the group. Finally, all quotes were translated from German to English and cross-checked. Study participants had the opportunity to comment on transcripts and/or ask for any corrections, but no feedback was provided. Since one OP withdrew their informed consent after the interview (of 1st round in 2018), the concerned material was excluded from analysis. The study participant did not indicate any reasons for the withdrawal of the transcript.

## 3. Results

The formative evaluation complementing the RCT took place in three waves, each one in 2018, 2019, and 2020. Interviews with OPs lasted approx. 20 to 40 min each (1st round: *n* = 10, 2nd round: *n* = 13), the interviews with ETTCs lasted approx. 20 to 60 min each (*n* = 9).

Study participants had diverse backgrounds and took different roles in the main study (see Table 1). All of them perceived the study work as an additional (and sometimes temporary) task within their main occupation.

### 3.1. Change Efficacy

#### 3.1.1. Situative Factors

The variety of employment tasks for ETTCs and OPs depends on different cost units and care facilities (e.g., company site, rehabilitation or training center). ETTCs are typically practice managers as well as employees of rehabilitation facilities specialized in occupational injuries. Their work has different focuses—for example, concerning testing procedures or therapy approaches. Most patients are referred to the facilities by prescription from physicians, but they can also train as free members, if there is an affiliated fitness center. Here, the main task of the ETTCs is to support members by providing assistance or corrections in exercise performance.

OPs provide advice to employees and employers on all aspects of occupational healthcare. In accordance with statutory requirements, these include preventive medical checkups, counseling sessions, occupational integration management, meetings, occupational health management, free consultation hours, employment examinations, second opinions on findings, and health promotion. Occasionally, general medical or family doctor tasks are also carried out for employees, but the law does not allow prescriptions or certificates of incapacity to work to be issued. Some of the OPs also operate their own emergency call service at the site or provide acute care for minor employee complaints, but most of them are directly employed by the company. If the OPs are employed by an intercompany service, the contractual partners are divided among the OPs employed. The distribution depends on the number of employees in the company. These can be employed in the administrative or technical area, as well as in production facilities.

Concerning the implementation of the study program, both OPs and ETTCs report limited time resources, since test and training plans as well as consultations are narrowly timed, often with negative impacts on personal relationship building with customers. Standardized procedures and schedules may be beneficial for structural and organizational tasks, especially in shift work. These general work conditions are sharpened by the additional study project: questionnaires, additional tests, and a higher communication load demand an advanced filing system to differentiate study participants from other customers. However, the high frequency of training allows for more personal contact, which is also necessary, since many of the study participants are training beginners. In general, ETTCs wish for more agency in treatment planning. OPs are happy to delegate some of the study tasks to case managers.

*“So, it’s like, I’m very, very overwhelmed with administrative tasks at the moment and my... number of patient contacts I have is very poor. (…) Well, the situation for my colleagues is a bit different. They have very few administrative tasks, but very many patient contacts.”*

(BA5 32)

*“Well, I see a number on the tablet that says ‘massage’. I finish that quickly and hand over the tablet and patient afterwards. Not very personal...”*

(MA6 16)

*“Because there’s no time for more. Motivation maybe…, but no time indeed.”*

(BA5 64)

*“There’s definitely no time to explain the study arms. In fact, I do not want to go into detail. I’ll leave that to the case managers.”*

(BA2 68)

#### 3.1.2. Resource Availability

The standardization of test procedures facilitates filling in by colleagues, in times of vacation, illness, shift work, or other duties. The same is true for training supervision, since the documentation of training progress follows a standardized protocol. If possible, neat support is desired. Trainers are identified as the most important contact person in the training phase. One-on-one supervision of trainees is rather rare, but can offer an advantage because it creates very close contact. While trainees like to have the same supervisor, there is also a benefit seen in new ideas for or influences on training with varying trainers. In some training centers, the workload can be reduced by delegating it to other staff. For example, students take on the task of monitoring weekly training attendance and sending this information to case managers. However, delegation also entails a loss of control. The training itself can take place independently of staff, but a trained staff member had to be present for the initial interview. In addition, enrollment staff had also to be instructed on the process for checking in and documenting participation via a signature list. They are then also responsible for billing health insurance companies, scheduling appointments with therapists and trainers, and contacting case managers. At the same time, all consent forms and data protection notices are processed here. Scheduling can be very complex in this regard: training centers often have to schedule around other practitioners’ appointments because patients have other healthcare appointments. At the same time, scheduling must remain flexible so that new patients can be referred to training in a timely manner.

Depending on the availability of colleagues, the OPs describe varying experiences regarding the importance of personal contact and depth of information availability. They are either jointly responsible in a team, or alone at the site. Within a team, there are also regular meetings among the OPs. One participating OP is not yet fully aware of the project, but has not been working at the site for long. However, informing the OPs about the intricacies of the project is rated as very important. The more patient contact the OPs have, the more important this information is. However, there is high turnover among OPs, which makes it difficult to ensure that all colleagues are kept informed. In some cases, the sites are poorly staffed, leaving little time for project work. If several OPs are responsible for a site, they have different levels of employee contact, depending on their area of responsibility. OPs, who have to do a lot of organizational work, have correspondingly less direct contact. In some cases, organizational work or appointment scheduling can also be delegated to receptionists. OPs have to deal with many different groups of people, who have to work together to ensure that processes run smoothly.

*“Well, I mean, there are a lot of interfaces, right? Well, there are interfaces with the human resources department, of course, there are interfaces with our health studios here, there are interfaces with the psychologists we have in-house, there are interfaces with the insurances. So, sure, it’s a network.”*

(BA5 44)

*“Exactly, that’s documented. We use digital solutions, but also old-school paper. The training follows standardization. Therefore, each trainer knows exactly what exercise at what device with what arrangements. If colleague A has designed the plan, colleague B can supervise the trainee without any problems. (…) The same is true for the general diagnostics and test. That makes collaboration easy.”*

(MA2 42)

*“There were critical times at some point when people said: Now it’s already very crowded here and the filing and work is overflowing and then I have to do another test. But basically, it’s such a personal feeling. You can never avoid that. But in terms of the time required, as we have also discussed, over the three years, I think it was almost, … So you can say that it was very bearable.”*

(MA3 2102)

#### 3.1.3. Task Demands

Most OPs have found their own solutions in cooperation with the case managers of the company health insurance funds that are aimed at their specific regional circumstances. This includes questions regarding general task assignment, but also which offices and which marketing tools could be used. The task definition between ETTCs and case managers is more pre-determined. Case managers are perceived as the key drivers of the project. Direct contact between OPs and ETTCs is rare. A major advantage for the study is that most case managers could build up on an already existing regional network. Benefits independent of the study success could be recognized. Facilitators are on-site offices, digital solutions for documentation, and possibilities to personally approach the employees, not via telephone. There is a lively exchange with the company health insurance funds on individual specialist areas—for example, psychological issues, for which the company health insurance funds provide experts. Cooperation with one’s own company health insurance fund is seen as positive, since there are special advantages for the patient. One OP, however, says that there are no points of contact with other groups of people outside his own company, but that there is all the closer cooperation within the site. Some described cooperation and regular meetings among the OPs within the same site. Additional cooperation comprises a wide variety of medical service providers, such as nutritionists, physiotherapists, and addiction counselors. Overall, the collaboration is described as good. Short distances to the company health insurance funds are perceived as an advantage: personal meetings with a direct contact person are rated better than pure telephone/email contact. Some OPs also have testing and training centers in the immediate vicinity, which can additionally reduce the distances that patients have to travel. In some companies, there are even in-house training facilities for the study participants, including gyms, swimming pools or other fitness offers.

Some OPs see their main task in the course of the study in the recruitment of employees. This includes identifying employees during preventive medical checkups or aptitude tests. Unlike case managers, OPs are thus able to assess suitability not only on the basis of documented diagnoses, but also on the basis of a personal assessment. It is also seen as an advantage that some OPs already know many of the employees and, in this context, can influence the decision even further—in particular, if an employee complains about long-term and serious MSDs. Some OPs feel responsible for raising awareness of the project among their patients. However, from the OPs’ perspective, the main recruitment tasks tend to rest with case managers.

Recruited study participants receive the contact details of the relevant test center from the case manager and independently contact the center to arrange a test appointment. The test usually takes three hours and involves functional testing of a shortened version, which is aimed primarily at restricted functions in the work context. Based on the test results, an individual training plan is set up for the employee.

*“We’re a rehab center, so it’s on prescription. It’s not a gym, it’s a real rehabilitation center via medical prescriptions and approvals, applications for rehabilitation, which are then carried out at our center. We have doctors in the center, we have psychologists, therapists, physiotherapists, masseurs. What else do we have? Nutritionists. So… the complete program of medical primary care providers… in principle.”*

(MA3 20)

*“We know our person in charge that we can contact, if we have any questions. The collaboration is smooth.”*

(MA5 74)

*“Let’s say it’s contacts via contacts. [...] So that means that if there are any sporting activities that can be offered, whether they are courses, whether they are lectures, etc., we are a bit in the same boat with the insurance company. That means we have larger companies around here [...]. At some point, inquiries were made as to whether we could offer any fitness courses from our premises, and then the talks came about bit by bit. That is, either it was the companies’ own initiative, or we got involved in the companies via the insurance company, which, let’s say, also got involved.”*

(MA7 28)

### 3.2. Change Commitment

#### 3.2.1. Process Quality

Recruitment is a major challenge for OPs. Although recurrent e-mails and reminders are sent by the case managers, the daily routine is often too busy to actively consider study participation for the employee seeking advice. Exchanges about recruitment measures, patient information, and successes are highly desirable, but difficult for various reasons. Nevertheless, some OPs meet with the case manager and discuss specific cases. Occasionally, case managers have special inquiries about certain employees and the extent to which they might be potential participants in the project. This means that the OPs check the case manager’s assessment before the initial contact and report back. For other OPs, there is lively contact regarding participants, recruitment, or even module recommendations. Even in the case of employees whose participation is still questionable, OPs and case managers consult with each other. Sometimes, the case managers only receive brief information about a consultation that has taken place, so that they can check later whether the employee has registered. However, this only happens with the explicit permission of the patient. Often, this information is then passed back to the OP. This exchange of information also occurs when the main recruiting task lies with the case manager and the OP is only informed about participants. A few OPs report that they have almost no exchange with case managers. The spatial separation of case managers, Ops, and companies is perceived as a barrier. Since ETTCs were only marginally involved in recruitment procedures, they had only assumptions about the recruitment success. Lacking motivation of companies, limited time resources of case managers, low motivation of employees, alternative healthcare programs, insufficient marketing, long distances, high training load, and shift work are assumed barriers for recruitment and the maintenance of training. Facilitators are positive experiences of co-workers and word-of-mouth-advertising. Longer training periods of six instead of three months were suggested to yield more sustainable results.

*“It is so often lost in the everyday life. No possibility to think about the study.”*

(BA5 72)

*“We had a very optimistic start, because that’s a nice project. (…) But well, our employees are the kind of people that say ‘someone else should fix that’—even in dealing with their own diseases.”*

(BA1 90)

Some of the OPs criticize the high additional workload that would not stand in relation to the financial compensation. On-site OPs have advantages over colleagues that work for several companies. In particular, the OPs’ attitude towards Module C (reintegration) lacks conviction. Module A (early prevention) is perceived as more persuasive (see [3,4] for detailed description of the modules). The thorough tests are laborious from time to time, but study participants feel well cared for. The project widens the competencies of the OPs, since they can initiate treatments without further consultation with general practitioners. Overall, the randomization design is perceived as difficult. For ETTCs, case managers serve as a key driving force in the project.

*“It’s the good work of the case manager! She really cares for their customers and leads them through the whole process—that’s a huge plus!”*

(MA4 96)

*“See, if there’s finally someone willing to participate, and then he only receives a … let’s say it offhand: a thera-band—he won’t like that. Enthusiasms looks different. I know that randomization is necessary for the quality of the study, but it’s not very helpful for the atmosphere.”*

(BA9 68)

OPs wish for more comprehensive feedback and information about the study status. Progress of individual study participants is complicated to retrieve, partially because of data protection regulations, and partially because of the lack of a digital solution for data transmission. Additional best-practice meetings would have helped them to exchange easy solutions to common problems.

#### 3.2.2. Quality of Results

Most of the ETTCs recognize study participants’ health benefits from baseline to follow-up. This leads to motivation also among colleagues. OPs are skeptical about the supported self-management group, since the yielded effects are assumed not to be sustainable. The OPs believe that better retention of participants in the company health insurance fund can keep dropout rates low through individual support and a fixed contact person, thus reinforcing the advantage of case managers and their responsibility for coordination. Compliance should also be increased by a higher financial contribution, a kind of “pledge,” which makes participation more binding and would be repaid after successful completion of the training period. Overall, the OPs assess the motivation of the employees as very variable. The control group seems to be generally dissatisfied and envious of the intervention group, which leads to even less initiative being shown here. In the intervention group, too, the employees should be more closely monitored in their progress by the case managers. ETTCs also report that participants who do not see immediate results doubt the overall concept. However, there are also very open and committed study participants who are willing to take the initiative and gladly accept assistance. The ETTCs describe the employees as very reliable overall, so that only very few tests are not taken in the test centers during the entire project period.

In the before-and-after comparison of patients’ symptoms, the ETTCs usually notice medium to strong improvements. In some cases, however, there are no changes at all. They have hoped that the project could create a certain awareness for functional training in relevant patients. In their opinion, long-term results can only be achieved in connection with extensive diagnostics. It has been mentioned that good results cannot always be recorded on paper: rather, it is a matter of the employee’s well-being, which in turn provides more motivation for further training.

The OPs tend to see an increase in success compared to other offers from the companies. They point out that there is always a risk of reverting to old habits after the project is completed. However, if the project were to continue in the long term, this could provide an opportunity for sustainability for the employees. The OPs report that when employees actually participate, they show success after a few weeks. Some have even been able to establish a long-term relationship: even after completion of the three months, the employees continued their training in the gym. Here, they again put into perspective the supposed lack of difference from previous prevention offers: the existing offers are more likely to be taken up by healthy employees, but now it is also possible to inspire other employees to participate.

*“We saw reliable results, also from the testing, and patients that the positive effects were highly motivated and diligent with the exercises. They even had fun, I think, it was a personal success for them.”*

(MA1 54)

*“This was of course very positive for the members or for the people who took part in the study, they were of course very strongly motivated by it and also had the corresponding success after this study. So you could clearly see that they were also improving from time to time.”*

(MA5 48)

*“But for the project itself, I really wouldn’t know. So, we have here, yeah, like I said, we’re out of the one control by now because it was just pointless. We didn’t have anything... was a lot of effort and didn’t come out of it.”*

(BA1 80)

#### 3.2.3. Other Outcomes

By participating in the project, ETTCs retrieve new ideas for other healthcare management programs, e.g., a newly launched physiotherapy consultation on-site at the companies. Further cooperation within the network, especially with the case managers of the company health insurance funds, are wanted and judged as likely to be continued also after study completion. Some training centers also hope that participation would increase their clientele: after the end of the training period, participants would ideally sign a subscription contract, which has not been realized in most of the training centers, yet. At some training centers, however, there has been a lot of interest in continuing the training, and membership was offered at a reduced rate at times. For the ETTCs, however, nothing has changed for their own actions or everyday work.

OPs acknowledge the high potential of occupational healthcare programs, since employee health has rapidly worsened in the last few years. From the point of view of the OPs, it would be advantageous to be able to continue to refer patients to physiotherapists—for example, without having to go via the family doctor or orthopedist. It would also be an asset if the project could lead to the empowerment of operating physicians not only in isolated regions, but throughout Germany, to be able to commission rehabilitation orders. Thus far, these requests have often been rejected by health insurers, since a job-related justification has not been sufficient. The willingness and awareness of the OPs for rehabilitation applications and measures could also be increased: they are more familiar with the application process and now advise the employee to take this path more often.

They see occupational health management as a means of encouraging employees to take care of their health, which has deteriorated rapidly in recent years. However, it would also have to lead to improved remuneration of employers for OPs in order to make such measures worthwhile in the future in comparison to the amount of work involved.

### 3.3. Context

#### COVID-19 Pandemic

The COVID-19 pandemic has caused changes in daily routines for most of the interviewees. Due to hygiene requirements, there have been limited capacities for tests and training. ETTCs and OPs observed that the introduction of compulsory masks had an effect on study participants’ motivation and adherence. Digital reservation systems are used to overview the current utilization rate and perceived as helpful. During the initial lockdown in March 2020, no testing or training was conducted at the testing and training centers due to legal quarantine requirements. Initially, testing was then allowed to continue if medically necessary, but the legal situation was unclear in the meantime. When the situation eased to some extent, the only differences from the usual working routine were the mask obligation and distance regulations, but most ETTCs mentioned that this had no influence on testing or training. The mask requirement also had no influence on motivation among the trainees. If necessary, some equipment had to be moved, or chairs in the waiting area removed, so that distance regulations could be fulfilled. The maximum number of people in a room has digitally been controlled by an automatic booking system, which meant that there was little effort on the part of the centers here as well. Contact with the case managers and the OPs also took place without any further restrictions. The main reason for an increase in workload was the constant introduction of new regulations and hygiene requirements. Some ETTCs as well as study participants were unsure about what was actually allowed and what was not. During the lockdown, additional employees were assigned short-term work, but this was subsequently remedied.

*“We closed the center for some time. Some of the study participants could not train for this period and it was hard for them to catch up with the training afterwards.”*

(MA2 48)

*“I would say that it still has a bit of an effect today that there are still people who have not been there since the reopening. They have simply said that it is too much for them with masks and here and there. So you can still see that, but the majority, I would say, of those who were always there, who also liked to come, they are also there again now. So in the meantime, we are back in the spheres where we were before.”*

(MA7 92)

## 4. Discussion

Good cooperation and communication between network partners is essential for the implementation of complex interventions. Our study delivered several aspects of readiness for change (with the key components of change commitment and change efficacy) perceived by different care providers and network partners in a multicenter RCT. Both groups perceived the case managers of the accompanying health insurance funds as key drivers in the regional and nationwide networks. This was partially driven by a top-down project leader attribution of case managers to fulfill the central role. Differential critical assessment of change by the care providers led to a missing collective change commitment and consequently to a missing organizational change commitment. The main inhibiting factors include lacking feedback about (e.g., recruitment) success, limited time resources of and narrow communication between responsible study staff, along with a low rate of utilization and limited adherence of the study population. The main facilitators include standardized procedures and documentation along with easy-access digital tools. Neither OPs nor ETTCs were fully convinced of the benefits of the envisioned change and showed skepticism about the necessary (personnel, time, and material) resources.

Overall, the OPs and the ETTCs were equipped with sufficient material resources to be able to meet the demands of the task on a collective level. The only hindering situational factor identified was limited time resources, which was additionally amplified by the lack of staff. Time limitations were mentioned disproportionately frequently in the interviews. Stress or lack of time have already been identified as indicators of a lack of change implementation skills [17]. Both OPs and ETTCs involved in the study had a varied daily work routine, with a heavy workload in the operational and administrative areas, and a strict schedule of appointments, often exceeding their regular working hours. This is particularly true for OPs who are part of an inter-company service and are further restricted in terms of time due to relocation and specific hourly quotas. For ETTCs, delegating work tasks can relieve some of the economic pressure. The recruitment of new study participants may involve less or more work for the OPs. Very detailed consultations and detailed descriptions of the study take considerably more time than simply raising the subject and sending it on to the case managers. Standardized workflows in the test and training centers enable a seamless transition of personnel. With the exception of testing, which requires certification, all employees were able to complete the necessary tasks. Further pre-identification of delegable tasks in future implementation projects could reduce the workload of the key players, especially considering the shortage of staff in some facilities. The high fluctuation among the OPs causes difficulties in the transfer of information, as new people are constantly being trained for the project and, at the same time, have to get used to a new working routine. High turnover and staff shortages can contribute to workforce demoralization and diminish the acceptance of change [17] and may also hinder delegation. The importance of access to knowledge and information was also noted by Miake-Lye et al. through their systematic review [18].

Most of the study participants have already been very strongly networked with companies, health insurance companies, and many other partners in their normal working routine and thus have extensive cooperation experience, especially in the context of corporate prevention. Both cooperative and informational lack of experience could be barriers to others. Overall, the service providers were well equipped and did not have to acquire additional resources to carry out their own tasks in the study. Structurally, however, long distances between the OPs, case managers, and ETTCs led to problems in communication among themselves and in the utilization behavior of the employees. Many researchers addressed structural conditions for assessing ORC—for example, human resources, technological infrastructure, and operational structures [18]. Digitalization makes it possible to simplify communication processes, but the prerequisites for this must be in place in the companies and practices.

The respective work processes were handled very differently by the individual stations due to different perceptions of one’s own area of responsibility and what this comprises. For example, some OPs took on a greater role in recruitment than others. Tasks that have to be taken on because of unclear role assignments, even though they do not lie within one’s own area of responsibility, form a barrier to implementation. Lack of clarity about responsibilities [18,19], or even disregard for one’s own responsibilities, as well as confusion about the overall process, can negatively impact the implementation of change [20]. A defined structure is particularly important, as interventions that require the cooperation of different providers increase the likelihood of miscommunication and delays in implementation.

The evaluation of costs and required resources due to workload, time, and stress factors has an influence on the acceptance of the intervention and is frequently cited as a reason for negative attitudes toward the measure [21]. The higher the individual’s self-efficacy, the more likely the person is to be willing to implement the changes [22]. It is particularly important to accurately communicate the reasons for the change and its benefits, i.e., in this study, to broadly communicate the added value for all key players compared to existing measures. Even if the overall organization has a positive attitude toward the changes, one’s own attitude toward the intervention can have an impact at the individual level: a perceived loss of control or obligation to implement the change may be some of the reasons here [21]. A positive organizational attitude toward the change, on the other hand, can transfer to organizational members and thus ensure that new changes are implemented more successfully [23].

From the OPs’ perspective, the problem with recruitment can be traced back, among other factors, to a lack of compliance on the part of the staff. For this reason, the study design with a self-management group represents an obstacle (also see [11]). Lack of perceived self-efficacy has already been identified in previous research as an inhibiting factor for the uptake behavior of innovations [24]. OPs report a lack of feedback on their own participant numbers, figures from the test and training centers, but also regional or Germany-wide key figures on the successes or failures of the study. Since the OPs perceived a lack of relevance as a result, they were less motivated. Although “best practice” models have been discussed in project team meetings and have also been addressed, more frequent feedback might have improved feelings of ownership and positively influenced confidence in the intervention process [17].

In addition, communication among the key players within the project tasks was also difficult. From the OPs’ perspective, data protection and medical confidentiality represent a particular hurdle. Mindfulness, respect, trust, and connectedness, as well as interdepartmental and organization-wide communication in team relationships, are critical components of change readiness [18]. Communication processes account for a particularly large proportion of barriers when the intervention requires cooperation between different staff or stations: lack of collaboration and communication can have a significant impact on this. Open and clear lines of communication can promote the implementation process. Especially among employees, interaction with other participating colleagues is particularly important [18]. Informal training and sharing information with each other provides better training than formal information sessions conducted prior to the start of a study [22]. ORC is often operationalized through structural attributes, individual attributes, and networks and communication, which highlights the important role of organizational structure, its people, and their collaboration in influencing readiness [18].

Change valence, or change worthiness, is often examined in the literature only from an individual perspective [22]. However, valence can also extend beyond individual benefits to the individual. In this case, both patient-related and health system aspects are mentioned here. Multiple perceived benefits can have a particularly strong influence on individual readiness: on the one hand, the study was able to initiate new projects and, on the other hand, it provided ideas for future projects. The specific possibility of raising employees’ awareness of their own health competence by putting prevention into practice in the workplace should also be emphasized.

The delivery of healthcare generally takes place in a complex adaptive system. Likewise, in this study, within such a large network with many specific regional characteristics for each site, there had to be a good balance between the clear distribution of roles with explicit task assignment and flexibility towards site-specific demands. Uncertainties about responsibilities [19] or a disregard of one’s own tasks diminish implementation success [20]. These are more likely to arise in case of demoralization due to a high fluctuation rate in staff or personnel shortage [17]. Access to relevant knowledge and recent developments—for example, by periodic training courses—should therefore be a standard to allow new members of the team to catch up with the team competencies. It is crucial to communicate the clear advantages of the change to make the implementation efforts plausible. If parts of the change are perceived as unfair (in case of low incentives or a work-overload, perceived loss of control, or high pressures), the low commitment of single individuals may sabotage the whole organization’s morale [21], whereas a generally positive organizational attitude may transmit to the individual level [23]. Standardization of procedures and documentation, along with collegial support and the possibility for consultations in cases of uncertainty, facilitate operating processes. Digital tools were rated as particularly helpful. Overall, all these factors show that the regional networks had a low readiness for change. Initial analyses and an organizational development plan accompanying the study implementation might have helped to build common goals and beliefs.

The found barriers and facilitators described by OPs and ETTCs are in line with feedback of recent analyses [11,12]. The regional and national network adapted to changing circumstances over time, some of the behaviors were not predictable, and found results were non-linear to the study inputs, i.e., concerning study design and operational outputs—typical characteristics found in highly complex and adaptive systems [25]. Since the collective change commitment in OPs and ETTCs can be described as low and the ORC is therefore not a shared team property, further analyses could conceptualize the unit of analysis on a lower level, e.g., the regional team at one study site. It should be noted that, in principle, organization members may also be wrong in their perception of the overall ORC, if their assessment of the organization implementation capability is too critical or benevolent. Other factors such as the integration of stakeholders [26], the reflection of aversive reasons of care providers [27], as well as finance strategies [28] should also be considered.

### Strengths and Limitations

The presented qualitative study adds to the understanding of the barriers and implementations given by a newly introduced program at very different network sites, especially in an RCT setting. One strength of the analysis is the integration of both the perspectives of OPs as well as ETTCs for the contextual analysis. It would have been worthwhile to include the case managers’ perspectives as key agents in the networks, too.

One limitation factor is that we only invited providers within the study network, since we addressed the implementation success of the new program. Therefore, we cannot rule out selection bias, i.e., the good material and structural equipment of study partners can also be attributed to self-selection: this suggests that it is not participation that leads to the implementation of changes, but rather the structures already in place that led to participation. Thus, the resource availability for other operational settings could be fundamentally different. Nevertheless, the resulting study sample was heterogenous enough to deliver information from small- to large-sized companies, different sectors of production and service, as well as rural and urban environments.

Another limitation of our analysis is that we only used telephone interviews with a short questionnaire on demographics, instead of combining it with standardized comprehensive questionnaires concerning different aspects of team collaboration. We used our design since it allowed us to react more openly to delivered facets, instead of pre-defining the nature of the bound barriers and facilitators. However, future trials might consider to use a combination of quantitative and qualitative measures to specifically assess ORC prior and during the implementation process. Results of validated scales such the Organizational Readiness for Implementation Change (ORIC) [29] or the Modified Texas Christian University—Director version (TCU-ORC-D) [30] could be compared through multi-informant approaches.

It is important to recognize that organizational members can also be wrong in their evaluation of ORC. This can occur, for example, by overestimating (or underestimating) the collective ability to implement. With an inferior design or lack of feasibility, no change, no matter how consistently used, will yield benefits [3]. Moreover, ORC does not guarantee that implementation will affect quality, safety, efficiency, or any other expected outcome. Implementation effectiveness is a necessary but not sufficient condition for achieving positive outcomes. Furthermore, ORC is not the only factor that can influence successful implementation processes. Other factors, such as stakeholder engagement in preparation for implementation [26], awareness of reasons for provider aversion to change, and strategies and funding support for implementation are highly relevant [28].

In addition, the time of measurement here is at the end of the study period. Some authors assume that a measurement of ORC can be applied especially at the beginning of the implementation process. This may yield key information that could be helpful in selecting actual implementation strategies in the health sector.

Nevertheless, the consistency with other studies within overarching RCTs [10,11,12] shows that at least important topic areas relevant for ORC could be identified that can further direct future (mixed-methods or triangulation) research.

## 5. Conclusions

Healthcare providers constantly need to adapt to changing demands and environments. ORC is perceived as an appropriate concept to assess organizational capacity to engage in implementing change in healthcare. Our study shows that even comparably brief telephone interviews can be effective to detect missing ORC in newly created networks. Future large RCTs might include room for interventions to counteract these specific barriers in the early stages.

As seen in our study, missing ORC can be a major barrier to implementation success. In the final analysis, there was no collective commitment to change or willingness to change in the organization as the network defined as all OPs and ETTCs. The attribution of a central role within a network (e.g., the case mangers’ role within the study) may increase the motivation of this group, but may also restrain other parties involved in the network. Based on the findings, researchers may improve the development of new intervention studies, especially in a randomized setting. Politicians and managers can use the findings to improve the organizational conditions of healthcare programs in company contexts.

## Figures and Tables

**Table 1:** Sociodemographic details of study participants.

	Questions	Answers
1st round OPs	How old are you?	min: 41 years, max: 62 years, mean: 53 years
	What is your gender?	female: 7, male: 3
	Do you work in full or parttime?	full time: 7, part time: 2, other: 1
	For how many years have you been working as OP?	min: 4 years, max: 40 years, mean: 17 years
	For how many years have you been working for this company?	min: 4 years, max: 30 years, mean: 11 years
	Have you been involved in the study from its start?	yes: 8, no: 2
	How many companies do you support?	min: 1, max: 44, mean: 10
	Do you have experience with studies?	yes: 7, no: 3
	How many hours per week do you work for the study?	min: <1, max: 3, mean: 1
	Is there a substitute physician for you?	yes: 9, no: 1
2nd round OPs	How old are you?	min: 40 years, max: 67 years, mean: 56 years
	What is your gender?	female: 8, male: 5
	Do you work in full or parttime?	full time: 8, part time: 2, other: 1, no comment: 2
	For how many years have you been working as OP?	min: 5 years, max: 31 years, mean: 17 years
	For how many years have you been working for this company?	min: 1.5 years, max: 31 years, mean: 11 years
	Have you been involved in the study from its start?	yes: 10, no: 0, no comment: 2
	How many companies do you support?	min: 1, max: 60, mean: 18
	Do you have experience with studies?	yes: 7, no: 4, no comment: 2
	How many hours per week do you work for the study?	min: <1, max: 2, mean: 1
	Is there a substitute physician for you?	yes: 9, no: 2, no comment: 2
ETTCs	In the study, our center is…	exclusively test center: 3, exclusively training center: 4, both: 2
	If applicable, if which part of the center do you work?	test center only: 4, training center only: 4, both centers: 1
	How many employees do work in your center?	min: 10 employees, max: 48 employees, mean: 28 employees
	How many of these are involved in the study?	min: 1 employee, max: 5 employees, mean: 3
	How old are you?	min: 25 years, max: 53 years, mean: 41 years
	What is your gender?	female: 2, male: 7
	What is your job description?	sports scientist/teacher, practice manager, trainer, physiotherapist
	Do you work in full or parttime?	full time: 9
	Do you have a leadership role?	yes: 6, no: 3
	For how many years have you been working for the center?	min: 2.5 years, max: 25 years, mean: 12 years
	Have you been involved in the study from its start?	yes: 9, no: 0
	How many companies do you support?	min: 1, max: 3, mean: 1
	How many hours per week do you work for the study?	min: 0.5, max: 10, mean: 3

## Data Availability

The datasets used and/or analyzed during the current study are available from the study group on reasonable request. Please contact the corresponding author.

## Trial Registration

The overarching parent study was registered at the German register for clinical trials (registration no.: DRKS00015120).

## Abbreviations

BA	interview with occupational physician
ETTC(s)	employees of test and training centers
MA	interview with employee of test and training centers
MSD	musculoskeletal disorder(s)
OP(s)	occupational physician(s)
ORC	organizational readiness for change
ORIC	organizational readiness for implementation change
RCT	randomized controlled trial
TCU-ORC-D	Texas Christian University—organizational readiness for change—Director version
